# Psychological determinants of spring festival homecoming: attitude ambivalence moderates the effect of subjective norms

**DOI:** 10.3389/fpsyg.2026.1760865

**Published:** 2026-04-13

**Authors:** Huanyan Xie, Jing Du, Xueni Zheng, Yuan Xiang

**Affiliations:** Economics and Management School, Wuhan University, Wuhan, China

**Keywords:** attitude ambivalence, homecoming behavior, subjective norms, theory of planned behavior, VFR travel

## Abstract

**Introduction:**

Grounded in the Theory of Planned Behavior (TPB), this study examines how subjective norms influence Spring Festival homecoming intention and subsequent homecoming behavior. TPB posits that attitudes, subjective norms, and perceived behavioral control are central determinants of intention and behavior, and suggests that the relative weight of these determinants may vary across decision conditions. Building on this framework, we consider attitudinal ambivalence as a key moderator of the normative pathway.

**Methods:**

Using two-wave survey data from 884 Chinese participants, we tested a moderated mediation model using structural equation modeling.

**Results:**

The results indicate that stay-local subjective norms negatively predict homecoming intention and actual behavior. Moreover, this indirect effect becomes stronger under conditions of high attitudinal ambivalence.

**Discussion:**

This study shows that attitudinal ambivalence shifts the relative influence of subjective norms in predicting intention and subsequent behavior, clarifying how normative pathways operate when evaluative guidance is conflicted.

## 1 Introduction

The Spring Festival homecoming refers to the large-scale movement of individuals who return to their hometowns during the Spring Festival holiday, typically for family reunions, after working or studying away from home. This phenomenon is deeply rooted in Chinese culture. It reflects strong place attachment, as many Chinese regard their hometowns as their “roots.” In recent years, scholars have shown growing interest in the homecoming phenomenon. For instance, Liu et al. (2024) explore the geographical and cultural meanings of “returning to hometown” through drawings and photo-elicitation interviews. Pearce (2012) offers a pioneering perspective by suggesting that an emotional attachment drives homecoming to one's former home as a meaningful place.

Examining the factors influencing homecoming behavior is an important focus of homecoming research. Existing studies have largely emphasized individuals' attitudes toward returning to hometown, with emotional attachment and place belonging commonly discussed as key affective foundations shaping these attitudes and, in turn, return decisions (Pearce, 2012; Li, 2014; Io, 2015). Implicit in much of this work, however, is the assumption that individuals hold relatively coherent and internally consistent attitudes toward homecoming. Yet this assumption may not always hold. Returning to hometown during the Spring Festival often evokes both positive and negative evaluations, including emotional connection on the one hand and financial costs, travel burdens, or social pressures on the other (Hung et al., 2013; Li, 2014). Such mixed evaluations constitute attitudinal ambivalence (Thompson et al., 1995). Despite acknowledging these mixed aspects, prior research has paid limited attention to how individuals make decisions when such conflicting evaluations coexist. As a result, we know relatively little about how homecoming behavior is shaped under conditions of attitudinal ambivalence. When attitudes are ambivalent, they may provide less clear guidance for action, and individuals may therefore rely more heavily on other determinants beyond personal evaluation, given that ambivalence is often associated with reduced attitude strength and weaker attitude–behavior consistency (Armitage and Conner, 2000). This possibility raises a broader question concerning how established behavioral frameworks account for decisions formed under evaluative conflict.

To address this question, it is useful to draw on the theory of planned behavior (TPB), which conceptualizes attitudes, subjective norms, and perceived behavioral control as central determinants of intention and behavior (Ajzen, 1991). In the context of Spring Festival homecoming, individuals' evaluations of returning to their hometown, perceived expectations from important others, and perceived constraints such as travel cost and time availability are central determinants of their intentions and behavior (Ajzen, 1991). However, TPB has generally been developed under the assumption that attitudes provide relatively clear and stable guidance. When attitudes are internally conflicted, it remains unclear whether the relative influence of these components remains constant or shifts toward greater reliance on social or contextual cues. In particular, the role of subjective norms may become more pronounced when personal evaluations lack internal consistency, consistent with the view that the predictive weight of TPB components can vary across individuals and decision conditions (Ajzen and Fishbein, 1972; Trafimow and Finlay, 1996; La Barbera and Ajzen, 2020).

Overall, the theory of planned behavior provides a useful framework for understanding homecoming decisions. However, it generally assumes that individuals' attitudes are relatively clear and internally consistent (Ajzen, 1991). Building on this premise, our study examines how attitudinal ambivalence conditions the influence of subjective norms on intention and behavior. In testing this model, our study makes three contributions. First, it contributes to the TPB literature by identifying attitudinal ambivalence as a boundary condition under which subjective norms become more influential in shaping intention and subsequent behavior. While TPB highlights the role of normative influence in guiding behavior, prior research has paid limited attention to whether and when evaluative inconsistency changes the strength of the normative pathway (Ajzen, 1991; La Barbera and Ajzen, 2020; Hagger and Hamilton, 2024). By showing that subjective norms exert stronger effects when individuals experience higher ambivalence, our findings refine understanding of how TPB-relevant normative processes operate under conditions of evaluative conflict (Thompson et al., 1995; Armitage and Conner, 2000). Second, this study contributes to the homecoming literature by highlighting the role of social influence in shaping return behavior. While previous research has predominantly focused on individuals' affective attachment and personal evaluations of returning to hometown, the social expectations embedded in close interpersonal networks have received comparatively less systematic attention (Backer et al., 2017; Hung et al., 2013; Io, 2015; Li, 2014). By demonstrating that subjective norms meaningfully predict both intention and subsequent behavior, particularly when attitudes are mixed, this study offers a more comprehensive account of the determinants of homecoming behavior. Third, the study contributes to research on attitudinal ambivalence by shifting attention to the behavioral consequences of ambivalence in a real-world context. Much of the prior work on ambivalence has examined its effects on attitude strength or attitude–behavior consistency (Thompson et al., 1995; Armitage and Conner, 2000). Our findings illustrate how ambivalent individuals rely more heavily on social cues in decision-making, thereby clarifying the behavioral mechanisms through which internal evaluative conflict shapes action.

## 2 Literature review and hypothesis development

### 2.1 The theory of planned behavior

The theory of planned behavior (TPB) provides a widely used framework for explaining and predicting individual behavior (Ajzen, 1991). According to TPB, behavioral intention is the most proximal predictor of behavior, and intention is shaped by three components: attitude toward the behavior, subjective norms, and perceived behavioral control. Attitude refers to an individual's overall evaluation of performing a specific behavior. Subjective norms capture the perceived social pressure from significant others to engage in or refrain from a behavior. Perceived behavioral control reflects individuals' perceptions of the ease or difficulty of performing the behavior. Together, these components influence intention, which in turn predicts subsequent behavior (Ajzen, 1991).

Although TPB conceptualizes these components as central determinants of intention and behavior, prior research suggests that their relative influence may vary across contexts and individuals (Ajzen and Fishbein, 1972; Trafimow and Finlay, 1996; Bosnjak et al., 2020; Baeli et al., 2022; Costa et al., 2022). Ajzen and Fishbein (1972) argued that some behaviors are more strongly guided by personal attitudes, whereas others are more influenced by social expectations. Empirical studies have similarly demonstrated that the predictive strength of attitudes and subjective norms differs depending on situational characteristics and individual orientations (e.g., Trafimow and Finlay, 1996). These findings imply that the balance among TPB components is not fixed, but may shift under certain conditions (Ajzen and Fishbein, 1972; La Barbera and Ajzen, 2020).

However, most TPB research has focused on the strength of attitudes, subjective norms, or perceived behavioral control as independent predictors, paying comparatively less attention to the internal consistency of attitudes themselves. TPB generally assumes that attitudes provide relatively clear evaluative guidance for behavior (Ajzen, 1991). Yet in many real-world decisions, individuals may simultaneously hold positive and negative evaluations toward the same behavior (Thompson et al., 1995; Armitage and Conner, 2000). Under such conditions of attitudinal ambivalence, the relative influence of TPB components may change. In particular, when personal evaluations lack clarity, individuals may rely more heavily on alternative sources of guidance, such as perceived social expectations, consistent with evidence that normative influence can be contingent on contextual and conditional factors (La Barbera and Ajzen, 2020; Hagger and Hamilton, 2024).

### 2.2 Subjective norms and homecoming behavior

Homecoming during the Spring Festival represents a socially embedded decision situated within close interpersonal networks. For individuals who work or study away from their hometowns, returning to their hometown is often closely tied to family obligations, emotional attachment, and culturally embedded expectations regarding reunion and belonging (Backer et al., 2017; Hung et al., 2013; Io, 2015; Li, 2014; Pearce, 2012). Prior research has emphasized the symbolic and relational meanings of home, highlighting emotional bonds, place attachment, and kinship ties as important drivers of return intentions (Io, 2015; Li, 2014; Pearce, 2012). In this context, homecoming is not merely a travel decision but a socially meaningful act embedded in family and community relationships (Hung et al., 2013; Li, 2014).

Given this strong social embeddedness, perceived expectations from significant others are likely to play an important role in shaping homecoming decisions. Within the TPB framework, such perceived social pressure is conceptualized as subjective norms (Ajzen, 1991). Subjective norms reflect individuals' perceptions of whether important others think they should perform a given behavior and the extent to which they feel motivated to comply with those expectations (Ajzen, 1991). In the present study, we focus on stay-local subjective norms—that is, perceived expectations from important others encouraging individuals to remain in their current city rather than return to their hometown. During the COVID-19 pandemic, public authorities encouraged individuals to reduce travel to mitigate health risks, and significant others often expressed concerns about safety and social responsibility, thereby reinforcing expectations to stay local. When individuals perceive that important others expect them not to return home, they may experience social pressure to refrain from homecoming behavior.

According to TPB, subjective norms influence behavior indirectly through behavioral intention (Ajzen, 1991). That is, when individuals perceive stronger social pressure to avoid returning to hometown, they are less likely to form the intention to return, which subsequently reduces the likelihood of actual homecoming behavior (Ajzen, 1991).

### 2.3 The moderating role of attitudinal ambivalence

Attitudinal ambivalence refers to the simultaneous presence of positive and negative evaluations toward the same object or behavior (Thompson et al., 1995). Rather than representing a neutral midpoint on an evaluative continuum, ambivalence reflects internal evaluative conflict (Thompson et al., 1995; Schneider and Mattes, 2021). A substantial body of research suggests that ambivalent attitudes tend to be weaker, less stable, and less predictive of behavior than univalent attitudes (Armitage and Conner, 2000; Jylhä et al., 2023). When individuals hold internally consistent attitudes, these evaluations provide relatively clear guidance for action. In contrast, when attitudes are ambivalent, individuals may experience difficulty determining how to act, as competing evaluations reduce decisional clarity (Thompson et al., 1995; Armitage and Conner, 2000).

Within the TPB framework, attitude is conceptualized as one of the key determinants of behavioral intention (Ajzen, 1991). However, if the clarity and strength of attitude are diminished under conditions of ambivalence, its predictive influence may correspondingly weaken (Armitage and Conner, 2000). When personal evaluations offer less definitive direction, individuals may turn to other sources of information to guide their decisions. In socially embedded contexts, such alternative sources often take the form of perceived expectations from significant others. Subjective norms, as defined in TPB, capture these perceived social pressures (Ajzen, 1991). Prior research has shown that the relative importance of attitudes and subjective norms may vary depending on situational and individual characteristics (Ajzen and Fishbein, 1972; Trafimow and Finlay, 1996). Building on this line of work, the present study proposes that attitudinal ambivalence represents a specific psychological condition under which normative influence becomes more prominent (La Barbera and Ajzen, 2020).

Moreover, psychological research indicates that evaluative conflict can generate uncertainty and discomfort, prompting individuals to seek external cues when forming judgments (Jonas et al., 1997; Thompson et al., 1995). In socially embedded decisions such as Spring Festival homecoming, perceived expectations from significant others represent a salient and accessible source of such guidance. Consequently, when attitudinal ambivalence is high, subjective norms may exert a stronger influence on intention formation. Because intention serves as the proximal predictor of behavior, this moderation is expected to extend to actual homecoming behavior through the mediated pathway (Ajzen, 1991). Taken together, we propose the following hypotheses:

Hypothesis 1a: Stay-local subjective norms negatively influence homecoming intention.
Hypothesis 1b: Homecoming intention positively influences homecoming behavior.
Hypothesis 1c: Homecoming intention mediates the relationship between stay-local subjective norms and homecoming behavior.
Hypothesis 2: Attitudinal ambivalence moderates the effect of stay-local subjective norms on homecoming intention, such that this effect is stronger when ambivalence is high than when it is low. Consequently, the indirect effect of stay-local subjective norms on Spring Festival homecoming behavior via intention is stronger at higher levels of ambivalence.

## 3 Research design and methods

### 3.1 Participants

Participants were recruited through the Credamo online platform in China. Credamo is a widely recognized data collection platform, and many studies using its data have been published in leading international journals. Because this study examined homecoming behavior, we screened participants to ensure that their current city of work or residence was not the same as their hometown. Before beginning the survey, participants were asked, “Is your current city of work or residence the same as your hometown?” Those who answered “yes” were excluded, whereas those who answered “no” proceeded with the survey. To improve data quality, the survey incorporated basic quality-control procedures, including attention checks and response-time screening to exclude inattentive responses.

A total of 1,109 participants completed the first-wave (T1) survey, and 884 of them also completed the second-wave (T2) survey, yielding a retention rate of 79.7%. An *a priori* power analysis was not conducted prior to data collection. Nonetheless, the final sample size exceeds commonly recommended thresholds for structural equation modeling, providing adequate power for hypothesis testing. The final sample consisted of 884 participants who completed both waves. In terms of education, 69.1% reported a bachelor's degree, 11.3% a master's degree, and 0.9% a doctoral degree; 12.8% held an associate degree, and 4.9% reported senior high school/secondary vocational education or below. Regarding occupation type, 40.7% worked in private enterprises, 21.3% in state-owned enterprises, 17.9% were students, 9.9% worked in public institutions, 5.6% in foreign-invested enterprises, 3.5% were civil servants, and 1.2% reported other occupations. In terms of vaccination status, 57.1% reported receiving three doses or more, 41.0% two doses, 0.5% one dose, and 1.4% were unvaccinated. For prior homecoming frequency, 57.7% reported a high frequency and 25.9% a very high frequency of returning to their hometown for the Spring Festival, whereas 12.0% reported a moderate frequency and 4.4% reported a low or almost no frequency. Overall, the sample included participants from diverse educational and occupational backgrounds.

### 3.2 Measures

All variables were measured on a five-point Likert scale (1 = strongly disagree, 5 = strongly agree) unless otherwise noted. Because all participants were Chinese, the scales were translated into Chinese by the first author and then revised by three co-authors to ensure both semantic accuracy and clarity of understanding.

#### 3.2.1 Stay-local subjective norms

Stay-local subjective norms were measured with two items adapted from Taylor and Todd (1995). We focused on stay-local norms because public authorities encouraged people to stay local during the 2022 Spring Festival and significant others often endorsed this advice due to concerns about safety and social responsibility, making stay-local normative pressure particularly relevant. The two items were: (1) “People who influence my behavior would think I should stay local during the Spring Festival,” and (2) “People who are important to me would think I should stay local during the Spring Festival.” These items capture perceived expectations from important others, consistent with the TPB definition of subjective norms as perceived social pressure regarding whether one should perform or refrain from a behavior (Ajzen, 1991). The scale demonstrated acceptable reliability (Cronbach's α = 0.736). Although the coefficient is modest, alpha is sensitive to the number of items and is typically lower for very brief scales, making this value reasonable for a two-item measure.

#### 3.2.2 Attitudinal ambivalence

To capture ambivalence toward homecoming in the COVID-19 context, we adapted items from Tong et al. (2020). Participants evaluated both positive evaluations of staying local (e.g., “Staying local during the Spring Festival reduces the risk of COVID-19 infection”) and negative evaluations of staying local (e.g., “Staying local disrupts my life plans”). Both subscales demonstrated acceptable reliability (positive evaluations: Cronbach's α = 0.731; negative evaluations: Cronbach's α = 0.777). Attitudinal ambivalence was then computed using the standard formula: ambivalence = (*P* + *N*)/2 – |*P* – *N*|.

Where *P* represents the mean score on the positive evaluation scale and *N* represents the mean score on the negative evaluation scale. Higher scores indicate stronger ambivalence.

#### 3.2.3 Intention to return to hometown

Homecoming intention was assessed with a single item adapted from Taylor and Todd (1995): “Do you have a strong intention to return to your hometown during the Spring Festival?” We acknowledge that using a single-item measure limits the assessment of internal consistency reliability and may therefore be less robust than multi-item scales. Nevertheless, the intention item was anchored to a concrete and clearly defined behavior within a bounded time frame, and it significantly predicted subsequent homecoming behavior measured at T2, providing evidence of criterion-related validity in the present context (Ajzen, 1991; Matthews et al., 2022).

#### 3.2.4 Homecoming behavior

Actual behavior was measured at T2 using a single binary item: “Did you return to your hometown during the Spring Festival?” (1 = yes, 0 = no).

#### 3.2.5 Control variables

Several control variables were included based on theory and prior evidence. First, perceived behavioral control (PBC) and prior homecoming frequency were controlled because both are conceptually consistent with the TPB framework: TPB posits that perceived behavioral control, together with intention, is directly relevant to behavioral performance, and past behavior is often considered an important predictor of subsequent intention and action (Ajzen, 1991). Second, vaccination status was controlled because research during COVID-19 suggests that vaccination-related information and factors are associated with individuals' engagement in preventive and travel-reduction behaviors, which are closely related to “stay-local” decisions (Andersson et al., 2021). Finally, education level and occupation type were included because socioeconomic and role-related factors shape individuals' mobility constraints and flexibility, which can in turn influence travel decisions and holiday mobility (Kattiyapornpong and Miller, 2009).

### 3.3 Procedure

Data were collected around the 2022 Spring Festival (January 25 to February 8, 2022), a period during which individuals in China actively decide whether to return to their hometowns. This timing enabled us to measure homecoming intention shortly before the holiday and to capture actual behavior afterward. The 2022 Spring Festival occurred in the context of the COVID-19 pandemic, during which public authorities encouraged many people to stay local to reduce travel-related risks. Such guidance likely intensified individuals' mixed evaluations of homecoming and also shaped the advice they received from close others, who may have encouraged staying local out of concerns for safety and social responsibility. Importantly, however, the evaluative conflict underlying homecoming decisions is not unique to the pandemic period; individuals often experience ambivalence toward returning to their hometown due to the coexistence of emotional attachment and practical constraints. Thus, the 2022 context provides a salient setting to observe how subjective norms operate when attitudes are ambivalent, while the theoretical mechanism examined in this study is expected to be relevant beyond the pandemic context.

Participants were recruited through the Credamo online platform, where eligible respondents were invited to participate in a research survey. All study materials were administered as an online questionnaire. Before starting the questionnaire, participants read an introductory statement describing the study purpose and assuring confidentiality and research-only use of the data; continuing with the survey indicated their consent to participate. We employed a two-wave design. At T1 (1 week before the Spring Festival), participants completed measures of stay-local subjective norms, attitudinal ambivalence, perceived behavioral control, homecoming intention, and control variables. The median completion time for the full questionnaire at T1 was approximately 167 s. At T2 (1 week after the Spring Festival), the same participants were invited via the platform to complete a follow-up survey and report their actual homecoming behavior. The median completion time for the full questionnaire at T2 was approximately 66 s. This time-lagged design separated predictors and the behavioral outcome, thereby mitigating potential common method bias. Additional statistical checks for potential common method bias are reported in the Section 3.4.

### 3.4 Statistical procedures

We conducted the analyses in three steps using Mplus 7. First, we performed confirmatory factor analysis (CFA) to evaluate the measurement model. Model fit was assessed using multiple indices, including CFI, TLI, RMSEA, and SRMR. As a supplementary diagnostic for potential common method bias, we also conducted Harman's single-factor test by entering all self-reported items into an unrotated exploratory factor analysis. The first factor accounted for 35.27% of the total variance, suggesting that common method bias was unlikely to be a serious concern. In addition, we estimated a one-factor CFA including all self-reported items, which showed poor fit (χ^2^ = 586.28, df = 20, RMSEA = 0.15, CFI = 0.74, SRMR = 0.12), providing convergent evidence against a substantial common method factor.

Second, we estimated the hypothesized structural relationships with structural equation modeling (SEM). All predictors and controls measured at T1 were included in the structural model, with homecoming behavior measured at T2.

Third, we tested the proposed moderated mediation by examining whether attitudinal ambivalence moderated the indirect effect of stay-local subjective norms on homecoming behavior via homecoming intention. To estimate the interaction effect, stay-local subjective norms and attitudinal ambivalence were mean-centered and their product term was included as a predictor of intention. Indirect effects were evaluated using bootstrapping with 5,000 resamples, and conditional indirect effects were computed at high and low levels of ambivalence (±1 SD). Statistical significance was determined by bias-corrected 95% confidence intervals that excluded zero.

## 4 Results

### 4.1 Descriptive statistics

As shown in Table 1, the means (SDs) for stay-local subjective norms and homecoming intention were 3.98 (0.73) and 3.74 (0.95), respectively. Spring Festival homecoming behavior was coded as a binary outcome, with 54% of participants returning home (*M* = 0.54, SD = 0.50). Descriptive statistics and correlations among the study variables are presented in Table 1.

**Table 1:** Means, standard deviations, and correlations for all variables.

Variable	Mean	SD	1	2	3	4	5	6	7	8
1. Subjective norm	3.98	0.73								
2. Ambivalence	1.72	1.46	−0.26^**^							
3. Intention	3.74	0.95	−0.09^**^	0.15^**^						
4. The Spring Festival homecoming	0.54	0.50	−0.28^**^	0.16^**^	0.44^**^					
5. Past behavior	4.04	0.78	−0.04	0.05	0.17^**^	0.19^**^				
6. Perceived behavior control	4.09	0.71	0.45^**^	−0.39^**^	−0.16^**^	−0.30^**^	−0.11^**^			
7. Vaccine	3.54	0.59	0.08^**^	0.01	−0.03	−0.06	0.04	0.07		
8. Education	4.88	0.74	−0.07^*^	0.07^*^	−0.02	−0.04	0.07	−0.04	0.05	
9. Occupation	3.50	1.74	0.14^**^	−0.02	0.07^*^	−0.15^**^	−0.08^*^	0.18^**^	−0.05	−0.10^**^

^*^p < 0.05;

^**^p < 0.01.

### 4.2 Measurement model

A confirmatory factor analysis was conducted to assess the measurement model. The model demonstrated acceptable fit to the data (χ^2^ = 1710.77, df = 28, CFI = 0.956, TLI = 0.927, RMSEA = 0.071, SRMR = 0.045). All standardized factor loadings were significant and exceeded 0.50.

### 4.3 Hypothesis testing

We tested the hypotheses using structural equation modeling in Mplus 7. First, we tested whether stay-local subjective norms predicted homecoming behavior indirectly via homecoming intention. As shown in Table 2, stay-local subjective norms negatively predicted homecoming intention (β = −0.116, *p* < 0.05), supporting Hypothesis 1a. In turn, homecoming intention significantly predicted homecoming behavior (β = 0.205, *p* < 0.001), supporting Hypothesis 1b. Bootstrapping analyses with 5,000 resamples indicated a significant indirect effect of stay-local subjective norms on homecoming behavior through intention [estimate = −0.024, 95% CI (−0.040, −0.008)], supporting Hypothesis 1c.

**Table 2:** Mediation hypotheses testing results.

Variable	Intention (T1)	The Spring Festival homecoming (T2)
	β (S.E)	β (S.E)
Intercept	4.199 (0.189)^***^	0.784 (0.184)^***^
Education	−0.027 (0.040)	−0.044 (0.021)^*^
Occupation	0.059 (0.019)^**^	−0.037 (0.008)^***^
Vaccine	−0.034 (0.055)	−0.027 (0.024)
Past behavior	0.200 (0.050)^***^	0.061 (0.019)^**^
Perceived behavior control	−0.197 (0.044)^***^	−0.090 (0.021)^***^
Subjective norm	−0.116 (0.047)^*^	−0.115 (0.020)^***^
Intention		0.205 (0.014)^***^
*R* ^2^	0.874^***^	0.174^***^
IND	−0.024 (95% CI [−0.040, −0.008])	

^*^p < 0.05;

^**^p < 0.01;

^***^p < 0.001.

We then examined whether attitudinal ambivalence moderated the indirect pathway from stay-local subjective norms to homecoming behavior via intention. Conditional indirect effects were estimated using bootstrapping with 5,000 resamples and were evaluated at high and low levels of ambivalence (±1 SD). As shown in Table 3, conditional indirect effect analyses revealed that the mediation effect of intention was significant when ambivalence was high [estimate = −0.038, 95% CI (−0.057, −0.019)] and not significant when ambivalence was low [estimate = 0.024, 95% CI (−0.007, 0.052)]. The difference between conditional indirect effects was significant [difference estimate = −0.062, 95% CI (−0.098, −0.028)], providing evidence of moderated mediation. These findings support Hypothesis 2.

**Table 3:** Moderated mediation effects results.

Path	Level of moderator	Indirect effect
Subjective norm → Intention → The Spring Festival homecoming	High attitudinal ambivalence	−0.038 [95% CI (−0.057, −0.019)]
	Low attitudinal ambivalence	0.024 [95% CI (−0.007, 0.052)]
Difference between high and low attitudinal ambivalence		−0.062 [95% CI (−0.098, −0.028)]

## 5 Discussion

The scale of homecoming travel is substantial. For instance, during the 2023 Spring Festival, a total of 1.595 billion passenger trips were made via railways, highways, waterways, and airlines in China alone. Beyond China, homecoming also represents a significant share of overall travel in many countries, such as Australia, the United Kingdom, and South Korea (Backer, 2012; Lee et al., 2004; Seaton and Palmer, 1997). In the context of the Spring Festival, homecoming decisions are embedded in close interpersonal relationships and are often accompanied by competing considerations such as emotional attachment and practical constraints. Building on the TPB, this study examined how stay-local subjective norms relate to subsequent homecoming behavior through intention, and how attitudinal ambivalence conditions the strength of this normative pathway. The results showed that (1) stay-local subjective norms reduced homecoming intention, which in turn predicted actual homecoming behavior measured at T2, and (2) this indirect effect was stronger when individuals experienced higher ambivalence, highlighting that ambivalence changes how social expectations translate into real behavior via intention.

### 5.1 Theoretical contributions

This study makes three contributions to the literature.

First, it contributes to the TPB literature by identifying attitudinal ambivalence as a boundary condition under which subjective norms become more influential in shaping intention and subsequent behavior. While TPB highlights normative influence in guiding action (Ajzen, 1991), prior research has paid limited attention to whether and when evaluative inconsistency amplifies the normative pathway. Although prior TPB research has suggested that the relative influence of attitudes and subjective norms can vary across contexts and individuals, these moderators have typically been conceptualized as contextual characteristics or relatively stable individual differences. Our findings indicate that when individuals hold mixed evaluations in the focal decision context, subjective norms exert stronger effects on intention and subsequent behavior. By identifying attitudinal ambivalence as a decision-level psychological condition that systematically shifts reliance toward normative cues, this study advances that line of research by specifying when normative influence becomes especially consequential. This pattern aligns with recent TPB research suggesting that normative influence is contingent on context and conditions (La Barbera and Ajzen, 2020; Conner and Norman, 2022; Hagger and Hamilton, 2024). Moreover, although the present study did not include a measure of attitude toward returning to hometown, it clarifies how TPB-relevant determinants operate together under evaluative conflict. Perceived behavioral control was significantly related to both intention and behavior, suggesting that perceived behavioral control jointly shapes homecoming decisions alongside subjective norms. Importantly, this boundary condition is demonstrated not only for intention formation but also for subsequent behavior measured at a later time point, strengthening the behavioral relevance of conditional normative effects. In this way, the study shows that attitudinal ambivalence shifts the relative influence of subjective norms in predicting intention and subsequent behavior, clarifying how normative pathways operate when evaluative guidance is conflicted.

Second, the study contributes to the homecoming literature by foregrounding the role of social influence in shaping return behavior. Prior work on homecoming and VFR (visiting friends and relatives) travel has predominantly emphasized affective attachment, belonging, and personal motivations (Li, 2014; Io, 2015; Gálvez-García et al., 2024). Our results show that stay-local subjective norms meaningfully predict both intention and subsequent behavior. This finding suggests that homecoming decisions should be understood not only as individual preferences, but also as socially embedded choices shaped by interpersonal expectations.

Third, the study contributes to research on attitudinal ambivalence by shifting attention to its behavioral consequences in a real-world setting. Much prior ambivalence research has focused on attitude strength or attitude–behavior consistency (Thompson et al., 1995; Armitage and Conner, 2000; Schneider and Mattes, 2021). By examining an actual behavior measured later, our findings demonstrate that ambivalence does not merely weaken evaluative guidance, it can redirect decision-making toward greater reliance on social cues. This provides clearer insight into the behavioral mechanism through which evaluative conflict shapes action.

### 5.2 Practical contributions

This study also offers practical implications for stakeholders seeking to support homecoming related decision making. First, for family members and close others, the findings suggest that their expressed expectations may carry greater weight when individuals feel ambivalent. Offering clear and consistent guidance and avoiding mixed or conflicting cues may help reduce uncertainty and support more stable intention formation. Second, for public agencies and community organizations, the results indicate that normative appeals that emphasize shared responsibility and family safety may be particularly influential among ambivalent individuals, especially when communicated through trusted social networks rather than purely informational messages. Third, for travel and mobility service platforms, the findings point to the value of tailoring communication to users' psychological states. Reassurance oriented and norm based cues may be more effective for highly ambivalent users, whereas feasibility focused information, including clear travel guidance and flexibility options, may be more helpful when ambivalence is low.

### 5.3 Limitations and future research

Although this study yields meaningful findings, several limitations should be acknowledged.

First, some constructs were measured using single-item scales. While Matthews et al. (2022) argued that single-item measures can be acceptable, they are often criticized for lacking reliability and validity. To improve measurement quality, future studies should consider multi-item scales. Second, the empirical model did not include a direct univalent measure of attitude toward returning home, which limited our ability to test the full TPB structure. Future research should incorporate a direct attitude measure to more comprehensively examine the roles of attitudes, subjective norms, and perceived behavioral control. Third, age and gender were not collected, limiting our ability to assess demographic heterogeneity in homecoming decisions. Fourth, the data were collected through self-reports. Although we attempted to minimize common method bias by gathering data at two time points and by using time-lagged behavioral measure, the possibility of bias remains. Future research should incorporate multiple data sources to strengthen validity. Fifth, homecoming behavior was operationalized as a binary outcome indicating whether participants returned to their hometown during the Spring Festival, which is appropriate for capturing behavioral occurrence but does not reflect potentially meaningful variations in travel timing, duration, or distance. Future research could use more fine-grained behavioral indicators to examine these dimensions. Finally, the study was conducted in the context of the Spring Festival and during the pandemic period. While the underlying mechanism—greater reliance on social cues under attitudinal ambivalence—should be relevant beyond this setting, future research should test the model in other cultural contexts and in non-pandemic periods, and should incorporate a direct measure of attitude toward returning to hometown to examine the full TPB structure more comprehensively.

## Data Availability

The raw data supporting the conclusions of this article will be made available by the authors, without undue reservation.
